# Effectiveness of Exosomes from Different Mesenchymal Stem Cells in the Treatment of Psoriasis: A Murine Study and Meta-Analysis of Experimental Studies

**DOI:** 10.3390/biomedicines13092093

**Published:** 2025-08-28

**Authors:** Yu-Chen Huang, Chao-Yuan Chang, Chun-Jen Huang

**Affiliations:** 1 Graduate Institute of Clinical Medicine, College of Medicine, Taipei Medical University, Taipei 11031, Taiwan; 2 Department of Dermatology, School of Medicine, College of Medicine, Taipei Medical University, Taipei 11031, Taiwan; 3 Department of Dermatology, Wan Fang Hospital, Taipei Medical University, Taipei 11031, Taiwan; 4 Department of Medical Research, Wan Fang Hospital, Taipei Medical University, Taipei 11031, Taiwan; 5 Department of Anesthesiology, School of Medicine, College of Medicine, Taipei Medical University, Taipei 11031, Taiwan; 6 Department of Anesthesiology, Wan Fang Hospital, Taipei Medical University, Taipei 11031, Taiwan

**Keywords:** exosomes, extracellular vesicles, mesenchymal stem cells, meta-analysis, psoriasis, systematic review

## Abstract

**Background/Objectives**: Psoriasis is a chronic systemic inflammatory disease. Evidence on the efficacy of different mesenchymal stem cell (MSC) exosomes for psoriasis remains limited. This study aimed to evaluate the therapeutic effects of different MSC exosomes in mitigating psoriasis. **Methods:** The efficacy of human placenta MSC (hPMSC) and human umbilical cord MSC (hUCMSC) exosomes was compared in an imiquimod (IMQ)-induced psoriasis murine model. A meta-analysis was performed to incorporate the results of studies using IMQ-induced psoriasis murine models to compare MSC exosome treatments (exosome group) with vehicle or no-treatment controls (control group). **Results:** In this murine study, both the hPMSC and hUCMSC exosomes showed better effectiveness in reducing epidermal thickness and skin tissue cytokines than controls, but no significant difference was observed between the two MSC exosomes. Seven studies were included in the meta-analysis. Clinical severity scores were significantly lower in the exosome group than in the controls (standardized mean difference [SMD]: −1.886; 95% confidence interval [CI]: −3.047 to −0.724). Epidermal thickness was significantly reduced (SMD: −3.258; 95% CI: −4.987 to −1.529). No significant differences were found in most skin cytokines between the groups, although tumor necrosis factor-α mRNA (SMD: −0.880; 95% CI: −1.623 to −0.136) and interleukin-17A protein levels (SMD: −2.390; 95% CI: −4.522 to −0.258) were both lower in the exosome group. Meta-regression revealed a greater improvement in clinical scores in studies using hUCMSC exosomes compared to other MSC sources (*p* = 0.030). **Conclusions:** hUCMSC exosomes have been studied more extensively than other MSC exosomes. MSC exosomes reduce clinical severity and epidermal hyperplasia.

## 1. Introduction

Psoriasis is a chronic, immune-mediated, systemic, inflammatory, and lifelong skin condition [1,2]. It is marked by the overproduction of keratinocytes and the infiltration of T cells, dendritic cells (DCs), macrophages, and neutrophils [1]. The development of psoriasis is primarily influenced by a combination of genetic predispositions, environmental factors, and abnormal immune responses [3]. Despite extensive research, the exact mechanisms behind the development of psoriasis are still not fully understood. Over the past 20 years, the interleukin (IL)-23/IL-17A pathway has been identified as a crucial factor in the pathogenesis of this immune-mediated inflammatory disease [4].

Mesenchymal stem cells (MSCs) are cells that can differentiate into various lineages and possess the ability to self-renew [5]. MSCs exhibit strong immunomodulatory and anti-inflammatory properties [6]. These cells can be isolated from numerous tissues, such as the umbilical cord, endometrial polyps, menstrual blood, bone marrow, and adipose tissue [5]. MSCs have become the predominant cell type used in regenerative medicine. Multiple studies have shown the effectiveness of MSC-based therapies in treating skin diseases like chronic wounds [7] and atopic dermatitis [8]. Additionally, there has been a growing interest in researching the relationship between MSCs and psoriasis [9,10]. However, several issues limit the broad implementation of MSC therapy in clinical settings. These issues include potential tumor development and the risk of virus and prion transmission following stem cell transplantation, immune compatibility concerns between donors and recipients, ethical considerations, and the high costs of production [11,12,13,14]. Therefore, continuous research and long-term monitoring are necessary to thoroughly assess the long-term outcomes of MSC therapy, including possible adverse effects [11].

Exosomes, which are spherical lipid bilayer vesicles ranging in size from 30 to 150 nm, contain distinctive biomolecules, such as membrane glycoproteins, lipids, and cell-specific proteins, along with various types of nucleic acids [15]. Exosomes play a role in intercellular communication by carrying different types of cargo and can modulate the immune response by interacting with immune effector cells in the presence of anti-inflammatory substances [16]. Increasing evidence suggests that exosomes are key mediators of the therapeutic benefits associated with MSCs [17,18]. Compared to MSCs, exosomes from MSCs offer several advantages, including non-immunogenicity, absence of infusion toxicity, and ease of access and storage, as well as a lack of tumorigenic potential and ethical issues [19].

Recent evidence has highlighted the potential of mesenchymal stem cell (MSC)-derived exosomes for treating psoriasis, including findings from in vitro studies [20,21], murine experiments [22,23,24,25,26,27], and a single human trial [28]. However, conclusive evidence supporting their therapeutic efficacy remains limited. In addition, different MSC sources were used across studies, and no direct comparisons have been made between exosomes derived from different MSC origins. To address these gaps, we first conducted a murine study comparing the effectiveness of exosomes derived from human umbilical cord MSCs (hUCMSCs) and human placenta MSCs (hPMSCs) in an imiquimod (IMQ)-induced psoriasis murine model. We then performed a systematic review and meta-analysis—incorporating our experimental results—to provide a more comprehensive assessment of MSC exosomes in mitigating psoriasis.

## 2. Materials and Methods

### 2.1. Animal Experiments

#### 2.1.1. Isolation and Purification of MSC Exosomes

Exosomes were isolated and purified from human placenta-derived MSCs (provided by Professor Yen-Hua Huang, Taipei Medical University, Taipei, Taiwan) and human umbilical cord-derived MSCs (provided by Bionet Therapeutic Corp., Taipei, Taiwan). The procedure involved harvesting the culture medium, followed by centrifugation. The supernatant was then collected, filtered, and subjected to ultracentrifugation (Beckman Coulter Optima L-90 K Ultracentrifuge; 100,000× *g*; 4 °C; 90 min; Type 50.2 Ti rotor, k-factor: 157.7; Beckman Coulter Inc., Brea, CA, USA) in accordance with established protocols [29,30]. The resulting exosome pellets were sequentially resuspended, pooled, ultracentrifuged, resuspended, and further purified using repeated ultracentrifugation steps. The top gradients’ fractions were collected, diluted, and centrifuged. Finally, the exosome pellets were resuspended and stored at −80 °C. The exosomes could be stored for up to 14 days without any significant changes in concentration (Online Resource Figure S1) and were utilized within the 2-week timeframe to maintain concentration stability.

#### 2.1.2. Sizing and Morphology Analyses of MSC Exosomes

The MSC exosome size distribution and concentration were analyzed using nanoparticle tracking analysis (NTA) with the ZetaView PMX 110 system from Particle Metrix (Holly Springs, NC, USA), following the manufacturer’s guidelines. The MSC exosome suspension was fixed, placed onto grids from Polysciences (Warrington, PA, USA), and allowed to dry; then, its morphology was observed via transmission electron microscopy (TEM) using the Hitachi HT-7700 instrument from Hitachi (Tokyo, Japan) [31].

#### 2.1.3. Marker Analysis of MSC Exosomes Using Immunoblotting Assays

MSC exosome markers, specifically CD9, CD63, and ALIX, were identified using immunoblotting assays. Calnexin was also identified to assess the purity of exosomes. The procedure involved extracting proteins from the MSC exosomes, which were then separated by electrophoresis and transferred onto nitrocellulose membranes from Bio-Rad Laboratories, Hercules, CA, USA [32]. The membranes were incubated with primary antibodies specific to CD9, CD63 (anti-CD9 antibody and anti-CD63 antibody; 20597-1-AP and 25682-1-AP from Proteintech, located in Rosemont, IL, USA), ALIX (anti-ALIX antibody, ab235377 from Abcam, based in Cambridge, UK), and Calnexin (anti-calnexin antibody, IRM041 from iReal biotechnology, Hsinchu, Taiwan). Bound antibodies were detected via chemiluminescence utilizing an ECL Plus kit from Amersham Bioscience, Buckinghamshire, UK.

#### 2.1.4. Animals and Protocol

Eight-week-old male Balb/c mice were obtained from the National Laboratory Animal Center in Taipei, Taiwan. These mice had unrestricted access to food and water and were maintained under a 12-h light/dark cycle. All animal care and experimental procedures adhered to the guidelines set by the U.S. National Institutes of Health (NIH), and the study protocol received approval from Taipei Medical University’s Institutional Animal Care and Use Committee (approval number: LAC-2023-0515).

Adult male wild-type Balb/c mice (8 weeks old) were randomly divided into 4 groups (*n* = 6 mice/group): sham group, imiquimod (IMQ) group, hPMSC exosome-treated IMQ group, and hUCMSC exosome-treated IMQ group. A psoriatic phenotype was induced by daily topical applications of 50 mg IMQ cream (5% Aldara cream; Ensign Laboratories Pty Ltd., Mulgrave, VIC, Australia) for 6 consecutive days (Days 1–6) [22,25]. The exosomes were dissolved in phosphate-buffered saline and topically applied daily for 7 days since Day 4. The dosages of hUCMSC exosome and hPMSC exosome were both 1 × 10^8^ particles in 25 µL PBS. One day after the final treatment (Day 11), all surviving mice were evaluated for psoriasis area and severity index (PASI) scores [22,25] and weighed. Anesthesia was administered via intraperitoneal injection of a Zoletil/Rompun mixture [Zoletil^®^ (tiletamine–zolazepam, Virbac, Carros, France) and Rompun^®^ 20% (xylazine hydrochloride, Bayer, Leverkusen, Germany)] at a dose of 40/10 mg/kg body weight. Following anesthesia, the mice were euthanized, and their skin was harvested and analyzed. The epidermal thickness was evaluated by histological analysis. Skin tissue cytokines were measured using enzyme-linked immunosorbent assays. The allocation of mice and outcome assessments were blinded.

#### 2.1.5. Psoriasis Area and Severity Index Scores of Animals

The PASI score, which involves scoring erythema, scaling, and skin thickening on a scale of 0 to 4, was used to evaluate the severity of dermatitis induced by IMQ, as previously reported [22,25]. The cumulative PASI score ranges from 0 to 12 and provides an overall measure of the severity of the condition.

#### 2.1.6. Histological Analysis

Formaldehyde-fixed skin tissue samples were embedded in paraffin wax, serially sectioned, and stained with hematoxylin and eosin (Sigma-Aldrich, Burlington, MA, USA). Epidermal thickness was observed under a light microscope (MoticEasyScan Pro 6; Motic Asia, Hong Kong, China). The thickness of the epidermis was subsequently measured using ImageJ software (Version 1.52e), a free tool provided by the NIH, USA (available online: https://imagej.net/ij/download.html (accessed on 1 August 2021)).

#### 2.1.7. Enzyme-Linked Immunosorbent Assay (ELISA)

Freshly frozen skin tissues were homogenized and centrifuged; then, the supernatants were collected [32]. To determine the cytokine levels in the skin tissues, TNF-α (DY410-05), IFN-γ (DY485), IL-6 (DY406), IL-17A (DY421), and IL-23 (DY1887) concentrations were measured using ELISA kits (all from R&D Systems, Emeryville, CA, USA).

#### 2.1.8. Statistical Analysis

A one-way analysis of variance was performed to compare the differences among the groups. The data are expressed as mean ± standard deviation. A *p*-value of less than 0.05 was considered statistically significant. The statistical analysis was conducted using GraphPad Prism 10 software (version 10.1.0 (316), 2023) for Windows.

### 2.2. Systematic Review and Meta-Analysis

Our systematic review and meta-analyses were conducted according to the Preferred Reporting Items for Systematic Reviews and Meta-Analyses statement 2020 [33]. This systematic review was registered on Prospero (CRD420251022811).

#### 2.2.1. Data Sources and Search Strategy

We searched the PubMed, Embase, Web of Science, and Scopus databases to identify relevant research studies published before 30 April 2025. The keywords “psoriasis,” “exosomes,” or “extracellular vesicles” were used without limitations. The full search strings are provided in Online Resource Table S1. We also reviewed the references of the identified articles and related reviews to include all relevant studies, regardless of language.

#### 2.2.2. Eligibility Criteria and Study Selection

Given the limited human trials found in the initial search, we also included murine experiments and in vitro model studies that met all the following criteria: (1) studies involving psoriasis patients, psoriasis murine models, or psoriasis cell models; (2) studies in which at least one study arm received MSC exosome treatment; and (3) studies with outcomes that included clinical skin severity, epidermal thickness, cytokine levels, or other related measures. The exclusion criteria were as follows: (1) studies in which no relevant outcomes were reported, and (2) studies from which appropriate data could not be extracted. Case reports and review articles were also excluded. Two reviewers (Y.C.H. and C.Y.C.) independently carried out the initial search, removing duplicate studies and evaluating the titles and abstracts of the retrieved articles. Any disagreements were resolved through consultation with a third reviewer (C.J.H.) to reach a consensus.

#### 2.2.3. Quality Assessment

The risk of bias for the murine experiments was evaluated using the Systematic Review Centre for Laboratory Animal Experimentation (SYRCLE) bias risk tool, which includes 10 items [34]. Based on this assessment, the studies were classified as having a low, high, or unclear risk of bias due to insufficient information. For the in vitro model studies, the Toxicological Data Reliability Assessment Tool (ToxRTool), an 18-point checklist, was utilized. The studies scoring less than 11 points or not fulfilling all the critical criteria were considered unreliable, those scoring between 11 and 14 points were deemed reliable with restrictions, and those scoring between 15 and 18 points were regarded as reliable without restrictions [35]. All assessments were conducted independently by two reviewers (Y.C.H. and C.Y.C.), with a third reviewer (C.J.H.) assisting in resolving any disagreements.

#### 2.2.4. Data Extraction and Definition

Patient characteristics, animals, psoriasis models, types of MSCs, sample sizes, treatment protocols of MSC exosomes, study outcomes, and major findings were tabulated (Table 1 and Table 2). The clinical severity scores, epidermal thicknesses, and levels of cytokines were also extracted (Online Resource Tables S2 and S3). Throughout this manuscript, we used the term “exosomes” to represent the exosomes, extracellular vesicles (EVs), and small extracellular vesicles (sEVs) referred to in all the included studies. However, we list the terms the authors used in their original studies in the table. Data were extracted from tables and article texts and then digitized using the online tool WebPlotDigitizer version 4.8 [36], which converts each plot into a set of accurate estimates of x and y coordinates using an automated extraction algorithm. If data were not available in the original publication, the corresponding authors were contacted to obtain their raw data.

**Table 1:** The characteristics of the included human and murine studies.

Author	Study Type and Inclusion Criteria	Age (Years)	Origin of MSC-Exo	Grouping and Sample Size	Route of Exo	Protocol	Dosage	Outcome Assessment ^a^	Major Findings
Meybodi et al. [28], 2024	Phase I/II trial Age > 18 years PASI score 3–10 Duration > 6 M	36.6 ± 8.07	Human adipose MSC-Exo	Exo 50: 4 Exo 100: 4 Exo 200: 4	Intradermal	Single dosage	50, 100, 200 μg/mL per cm^2^	3 M A, B, C	A single dose of 200 μg significantly improved clinical symptoms and regulated inflammatory and anti-inflammatory markers.

Exo, exosome; M, month; MSC, mesenchymal stem cell; PASI, psoriasis area and severity index. ^a^ Outcomes: A, skin severity scores; B, epidermal thickness; C, cytokines at the RNA level.

**Table 2:** The characteristics of the included murine studies.

Author	Psoriasis Model	Animals	Origin of MSC-Exo	Grouping and Sample Size	Route of Exo	Protocol	Daily Dosage/Mouse	Outcome Assessment ^a^	Major Findings
Rodrigues et al. [23], 2021	IMQ 6 D	8–12 weeks, C57BL/6 mice	Human UCB-MNC-sEVs	IMQ: 6 IMQ+vehicle: 6 IMQ+UCB-MNC-sEVs: 6	Topical (hydrogel)	IMQ D1–6 sEVs one hour after IMQ for D1–6	3 × 10^9^ particles/cm^2^	D 7 A, B, C, E	UCB-MNC-sEV significantly prevented or reversed acanthosis in IMQ-induced psoriasis and tendentially increased the number of Tregs in the skin.
Zhang et al. [22], 2021	Expt1: IMQ 6 D Expt2: IMQ 3 D	6–9 weeks, Balb/c male mice	Immortalized E1-MYC 16.3 human ESC-MSC-Exo	Expt1 IMQ+vehicle: 10 IMQ+MSC-Exo: 10 Expt2 IMQ+vehicle: 10 IMQ+MSC-Exo: 10	Topical (cream)	Expt1: IMQ D1–6+ Exo D4–6 Expt2: IMQ D1–3+ Exo D4–10	100 µg/mL, 200 µL	Expt1 D7 Expt2 D11 A, D	MSC-Exo resulted in reduced C5b-9 and IL-17 in a mild model of psoriasis.
Xu et al. [27], 2022	IMQ 4 D	6–8 weeks, C57BL/6 female mice	Mouse bone marrow MSC-sEVs	IMQ: 6 IMQ+MSC-sEVs: 6 IMQ+MSC-sEVs@PD-L1: 6 IMQ+MSC-sEVs@PD-L1+anti-PD-L1: 6	Intravenous	IMQ D1–4+ sEVs D1–4	50 µg	D5 A, B, C, E	MSC-sEVs-PD-L1 inhibited acanthosis, parakeratosis, and thickening of the stratum corneum and suppressed the inflammatory response by reducing immune cell infiltration, altering their phenotype, activating immunoregulatory cells, and regulating inflammatory cytokines in the skin and peripheral circulation.
Zhang et al. [24], 2022 (a)	IMQ 6 D	8 weeks, C57BL/6 female mice	Human umbilical cord MSC-Exo	IMQ: 6 IMQ+PBS: 6 IMQ+MSC-Exo: 6	Subcutaneous	IMQ D1–6+ Exo D0, D2, D4	50 µg	D7 A, B, D	MSC-Exo ameliorated psoriasis-like skin inflammation in mice by regulating the expression of IL-23 and IL-17 and inhibiting the maturation and activation of DCs.
Zhang et al. [25], 2022 (b)	IMQ 6 D	6–9 weeks, Balb/c male mice	Human umbilical cord MSC-IFNγ-sEVs	IMQ+PBS: 5 IMQ+ MSC-IFNγ-sEVs: 5 IMQ+ASO210: 5 IMQ+ MSC-IFNγ-sEVs+ASO210: 5 IMQ+ MSC-IFNγ-sEVs@ASO210: 5 IMQ+Hal: 5	Intradermal	IMQ D1–6+ sEVs D3–6	25 mg/kg	D7 A, B, C, E	IFNγ-MSC-sEVs reduced thickness, erythema, and scales of skin lesions, exhausted Th17 cells, increased Th2 cells, and reduced inflammatory cytokines. IFNγ-MSC-sEVs significantly improved the delivery efficiency and stability of ASO-210.
Zhou et al. [26], 2025	IMQ 6 D	8 weeks, C57BL/6 female mice	Human umbilical cord MSC-EVs	IMQ+PBS: 4 IMQ+nor-NOHA: 4 IMQ+MSC-EVs: 4 IMQ+nor@MSC-EVs: 4 IMQ+anti-IL17A: 4	Intravenous	IMQ D1–6+ EVs D1, 3, 5	100 μg	D7 A, B, C, E	MSC-EVs and nor@MSC-EVs displayed notable improvements in skin lesions and a substantial decrease in Ki 67+ cells. nor@MSC-EVs were the most efficacious therapeutic option for mitigating psoriasis.
Huang et al.	IMQ 6D	6–9 weeks, Balb/c mice	Human umbilical cord MSC-Exo Human placenta MSC-Exo	IMQ+vehicle: 6 IMQ+MSC-Exo (P): 6 IMQ+MSC-Exo (U): 6	Topical (PBS)	IMQ D1–6+ Exo D4–10	1 × 10^8^ particles in 25 µL PBS	D11 A, B, C, D	The hPMSC and hUCMSC exosomes both showed better effectiveness in reducing epidermal thickness and skin tissue cytokines than the controls, and no significant difference was observed between the two MSC exosomes.

ASO210, antisense oligonucleotides of miR-210; D, day; Exo, exosome; IL17A, interleukin 17A; IMQ, imiquimod; IFNγ, interferon-γ; M, month; MNC, mononuclear cell; MSC, mesenchymal stem cell; nor@MSC-EVs, mesenchymal stem cell-derived extracellular vesicles loaded with nor-NOHA; P, placenta; PBS, phosphate-buffered saline; PD-L1, programmed cell death-ligand1; sEV, small extracellular vesicle; sEVs@ASO210, sEVs loaded with ASO210; sEVs@PD-L1, sEVs loaded with PD-L1; Th, T helper cell; Treg, T regulatory cell; U, umbilical cord; UCB, umbilical cord blood. ^a^ Outcomes: A, skin severity scores; B, epidermal thickness; C, cytokines at the RNA level; D, cytokines at the protein level; E, others.

#### 2.2.5. Data Synthesis and Statistical Analysis

Based on the outcomes, the effectiveness of MSC exosomes was compared to the vehicle controls and positive controls. Meta-analyses were performed before and after incorporating the present study when at least 2 studies provided the same outcome. Continuous variables were expressed by the standardized mean difference (SMD) with 95% confidence intervals (CIs). Data heterogeneity was assessed using the *I*^2^ test [37]. A fixed-effects model was used when *I*^2^ was less than or equal to 50%, and a random-effects model was used when *I*^2^ was more than 50%. Meta-regression was performed to compare exosomes from different MSC sources. Publication bias was evaluated using a funnel plot and Egger’s regression test if more than 10 studies were available [38]. Comprehensive Meta-Analysis Version 3 (Biostat, Inc., Englewood, NJ, USA) was used to perform all analyses.

## 3. Results

### 3.1. Experimental Results

#### 3.1.1. Confirmation of MSC Exosomes

**Figure 1:**
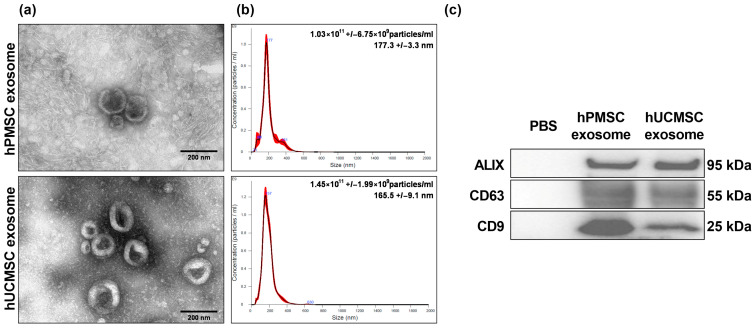
Confirmation of human placenta mesenchymal stem cell (hPMSC) exosomes and human umbilical cord mesenchymal stem cell (hUCMSC) exosomes. (**a**) Representative transmission electron microscopic images (60,000×) of MSC exosomes. (**b**) Sizing analysis of MSC exosomes by nanoparticle tracking analysis. (**c**) Representative gel photography of MSC exosome markers CD9, CD63, and ALIX, detected using an immunoblotting assay.

The MSC exosomes were verified based on three characteristics. They had a distinct double-layer cup-shaped morphology, as detailed in Figure 1a. Their particle sizes ranged from 150 to 200 nm, and the concentration was around 1 × 10^11^ particles/mL as shown in Figure 1b. Additionally, they were positive for the CD9, CD63, and ALIX markers, as outlined in Figure 1c. Both hUCMSC and hPMSC exosomes were absent of Calnexin (Online Resource Figure S2).

#### 3.1.2. hUCMSC Exosomes Improved Clinical Severity Scores, and Both MSC Exosomes Reduced Epidermal Thickness in the IMQ-Induced Psoriasis Murine Model

**Figure 2:**
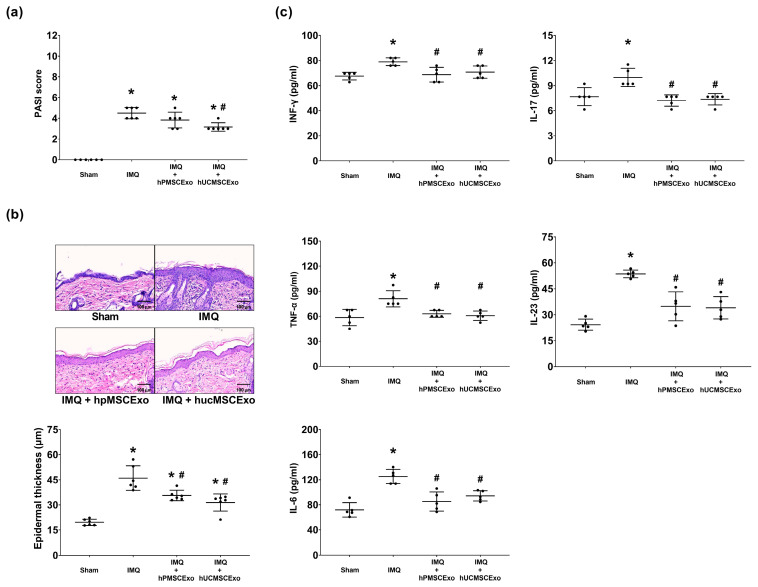
The effectiveness of human placenta mesenchymal stem cell (hPMSC) exosomes and human umbilical cord mesenchymal stem cell (hUCMSC) exosomes in imiquimod (IMQ)-induced psoriasis-like dermatitis in mice. (**a**) The psoriasis area and severity index (ranged from 0 to 12) (*n* = 6 per group). (**b**) Representative microscopic images of skin tissues stained with hematoxylin–eosin (400× magnification) and measurements of epidermal thickness (*n* = 6 per group). (**c**) Skin tissue cytokine concentrations, including interferon-γ (IFN-γ), tumor necrosis factor-α (TNF-α), interleukin-6 (IL-6), IL-17, and IL-23, were measured using enzyme-linked immunosorbent assays (*n* = 5 per group). All the data were assessed on the 11th day after IMQ. The data are presented as mean ± standard deviation. * *p* < 0.05 vs. Sham group; # *p* < 0.05 vs. IMQ group. Sham: Sham group; IMQ: imiquimod group; IMQ+hPMSCExo: hPMSC exosome-treated IMQ group; IMQ+hUCMSCExo: hUCMSC exosome-treated IMQ group.

On Day 11, the PASI scores were significantly lower in the hUCMSC exosome-treated IMQ group compared to the IMQ group (*p* = 0.001), while no significant difference was observed between the hPMSC exosome-treated IMQ group and the IMQ group (*p* = 0.139) (Figure 2a). Notably, epidermal thickness was significantly reduced in both the hUCMSC and hPMSC exosome-treated IMQ groups compared to the IMQ group (*p* < 0.001 and *p* = 0.007, respectively) (Figure 2b). However, no significant difference was observed between the two exosome-treated IMQ groups (*p* = 0.453).

#### 3.1.3. Both the hUCMSC and hPMSC Exosomes Mitigated IMQ-Induced Cytokine Upregulation in Mouse Skin Tissues

Consistent with the epidermal thickness findings, all measured cytokine levels were lower in the hUCMSC and hPMSC exosome-treated IMQ groups compared to the IMQ group. Specifically, the interferon-γ (IFN-γ) levels were reduced (*p* = 0.009 and 0.038), as were the tumor necrosis factor-α (TNF-α) (*p* = 0.023 and 0.009), interleukin-6 (IL-6) (*p* < 0.001 and 0.004), IL-17A (*p* = 0.001 and 0.002), and IL-23 (both *p* < 0.001) levels (Figure 2c). However, no significant differences in the cytokine levels were observed between the two exosome-treated groups (all *p* > 0.05).

Data from this animal experiment were included in the systematic review and meta-analysis.

### 3.2. Systematic Review and Meta-Analyses

#### 3.2.1. Search Results and Trial Characteristics

**Figure 3:**
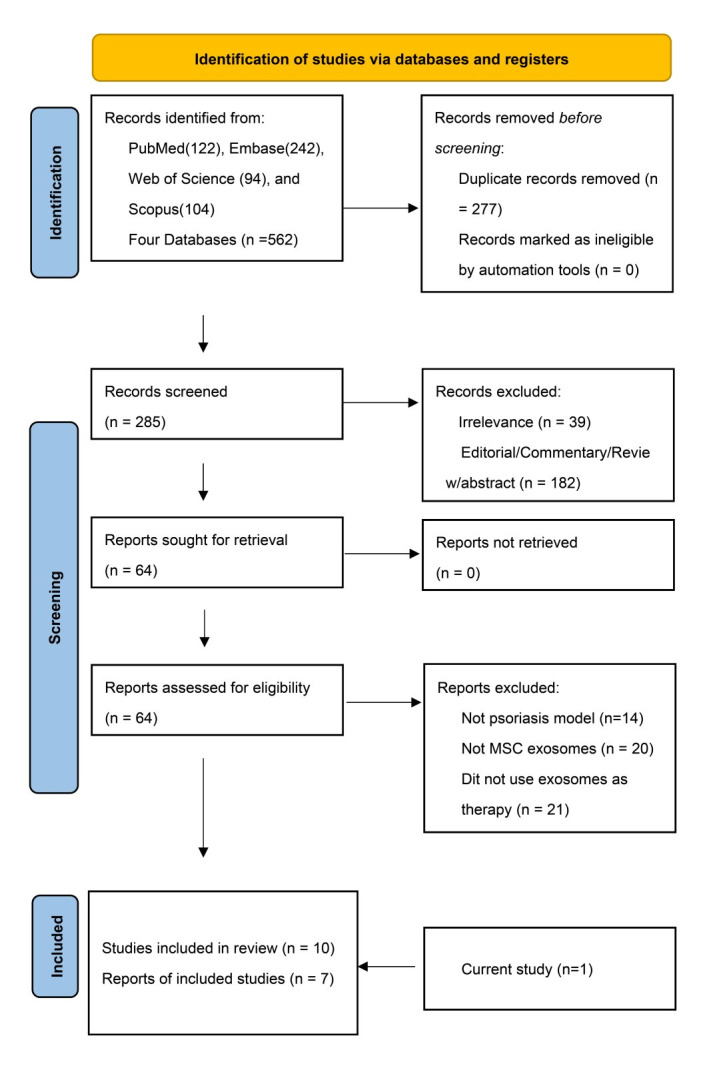
Flow diagram for selecting the included studies.

The literature search process is summarized in Figure 3. Of the 273 articles identified, 9 were selected after abstract screening and full-text review. Including our own animal study, a total of 10 studies were analyzed. Among these, one was a human open-label study [28], two were murine experiments (including the present study) [22], and two employed in vitro models [20,21]. The remaining five studies combined both murine and in vitro experiments [23,24,25,26,27].

Table 1, Table 2 and Table 3 summarize the main characteristics of the included studies. All of the murine studies applied IMQ topically to induce psoriasiform dermatitis [22,23,24,25,26,27]. The in vitro models primarily involved HaCaT keratinocytes stimulated by psoriasis serum-derived exosomes [20], IL-17A [24], or TNF-α combined with IL-17A [26] to mimic psoriatic conditions.

**Table 3:** The characteristics of the included in vitro studies.

Author	Cell Model	Origin of MSC-Exo	Dosage	Effect of Exo
Rodrigues et al. [23], 2021	THP-1 cells Human PBMCs	Human UCB-MNC sEVs	10^10^ particles/mL	UCB-MNC-sEV were shown to shift macrophages toward an anti-inflammatory phenotype, which, in turn, exerted paracrine effects on fibroblasts. The incubation of PBMCs with UCB-MNC-sEV resulted in reduced total CD4+ and CD8+ T cell proliferation and cytokine release while specifically supporting the development of Treg by influencing FOXP3 expression.
Xu et al. [27], 2022	LPS-treated BMDMs and BMDCs of mice AntiCD3/CD28-treated T cells from mouse lymph nodes	Mouse bone marrow MSC-sEVs and MSC-sEVs@PD-L1		MSC-sEVs@PD-L1 altered the phenotype of various activated immune cells to an immunosuppressed state and inhibited inflammatory cytokine production. MSC-sEVs@PD-L1 resulted in T cell anergy, Treg induction, and effector T cell elimination.
Zhang et al. [24], 2022 (a)	BMDCs of mice IL-17A-treated HaCaT cells	Human umbilical cord MSC-Exo	2.5 μg/mL	Co-cultured with Exo, the maturation and activation of DCs were suppressed, and the expression level of IL-23 was decreased. Exo suppressed IL-23 and CCL20 secretion of HaCaT cells by inhibiting STAT3 activity.
Zhang et al. [25], 2022 (b)	Human PBMC CD3+ T cells from PBMCs	Human umbilical cord MSC-IFNγ-sEVs and MSC-IFNγ-sEVs@ASO210		IFNγ-sEVs inhibited the proliferation and activation of PBMCs and T cells.
Kim et al. [20], 2023	Psoriasis serum-derived Exo-treated HaCaT cells	Human ADSC-Exo	3.7 × 10^9^/mL	ADSC-Exo suppressed pro-inflammatory cytokine and oxidative stress production and restored autophagy in HaCaT cells treated with psoriasis serum-derived exosomes.
Abed et al. [21], 2024	HUVECs	MSC-Exo	100 μM/mL	The concentration of the TGF-β2 gene in the target cells significantly increased following treatment with Exo.
Zhou et al. [26], 2025	TNF-α- and IL-17A-treated HaCaT cells BMDCs of mice Splenic cells isolated from IMQ-induced mice	Human umbilical cord MSC-EVs and nor@MSC-EVs	50 μg/mL	nor@MSC-EVs mitigated the psoriatic phenotype by inhibiting the expression of associated antimicrobial peptides, chemokines, cytokines, and inflammatory proteins, as well as those related to polyamine production and cell proliferation. MSC-EVs and nor@MSC-EVs directly inhibited the maturation of BMDCs and the differentiation of Th1 and Th17 in vitro, exerting direct immunomodulatory effects.

ADSC, adipose-derived stem cell; ASO210, antisense oligonucleotides of miR-210; BMDCs, bone marrow-derived dendritic cells; BMDM, bone marrow-derived macrophage; Exo, exosome; HUVEC, human umbilical vein endothelia cell; IFN, interferon; IL, interleukin; IMQ, imiquimod; IFNγ-sEVs, INFγ stimulated sEVs; LPS, lipopolysaccharide; MNC, mononuclear cell; MSC, mesenchymal stem cell; nor@MSC-EVs, mesenchymal stem cell-derived extracellular vesicles loaded with nor-NOHA; PBMC, peripheral blood mononuclear cell; PD-L1, programmed cell death-ligand1; sEV, small extracellular vesicles; sEVs@ASO210, sEVs loaded with ASO210; sEVs@PD-L1, sEVs loaded with PD-L1; Th, T helper cell; Treg, T regulatory cell; TNF, tumor necrosis factor; UCB, umbilical cord blood.

Various MSC-derived exosome types were utilized across the studies. The human study applied exosomes from human adipose-derived MSCs [28]. Among the preclinical studies, four used exosomes from human umbilical cord-derived MSCs (hUCMSCs) [24,25,26] or umbilical cord blood mononuclear cell-derived MSCs [23], while the remaining three used exosomes from mouse bone marrow MSCs [27], human adipose-derived MSCs [20], and human embryonic stem cell-derived MSCs [22]. Our study used both hUCMSC and hPMSC exosomes. One study did not specify the MSC source [21]. Additionally, three studies incorporated MSC exosomes loaded with specific therapeutic cargos: antisense oligonucleotides targeting miR-210 (ASO210) [25], programmed cell death-ligand 1 (PD-L1) [27], and an arginase-1 inhibitor (nor-NOHA) [26].

#### 3.2.2. Results of Quality Assessment

Online Resource Tables S4 and S5 summarize the quality assessment results. All of the murine studies [22,23,24,25,26,27] lacked sufficient details on random sequence generation, allocation concealment, and blinding of the interventions, making proper evaluation of these domains impossible. As a result, all murine studies were rated as having an unclear risk of bias. In contrast, four of the in vitro studies [20,23,24,26] were deemed reliable without restrictions. The remaining in vitro studies [21,25,27] were considered unreliable due to critical omissions—specifically, the failure to report the concentration of exosomes [25,27] or to specify the type of exosomes [21]—both of which are classified as red criteria in the ToxRTool assessment.

#### 3.2.3. Systematic Review

The human study included 12 patients who received a single intradermal dose of MSC-derived exosomes at varying concentrations (50, 100, and 200 μg/mL per cm^2^). The authors concluded that a single dose of 200 μg/mL per cm^2^ significantly improved clinical symptoms and modulated both pro- and anti-inflammatory markers [28].

Due to heterogeneity among the in vitro models using different types of cells, meta-analyses were not performed for the in vitro studies. Several studies demonstrated that exosomes derived from hUCMSCs [23,25] and mouse bone marrow-derived MSCs [27] inhibited the proliferation of human peripheral blood mononuclear cells (PBMCs) [25] and T cells [23,25,27] while promoting the induction of regulatory T (Treg) cells [23,27]. hUCMSC exosomes were also shown to suppress the activation and maturation of DCs [24,26] and reduce inflammatory cytokine production [20,23,24,26]. In the study by Rodrigues et al., exosomes derived from human umbilical cord blood mononuclear cells promoted macrophage polarization toward an anti-inflammatory phenotype, which subsequently exerted paracrine effects on fibroblasts [23]. Kim et al. reported that exosomes from human adipose-derived MSCs attenuated oxidative stress and restored autophagy in HaCaT cells [20]. Abed et al. observed increased expression of the transforming growth factor-β2 gene in human umbilical vein endothelial cells following MSC exosome treatment [21].

In contrast, the murine studies offered more comprehensive insights into clinical skin severity, epidermal thickness, and cytokine expression in skin tissues, and flow cytometry data. Meta-analyses were feasible for most outcomes, except flow cytometry. Among the seven murine studies, five (including the present study) [24,25,26,27] reported significantly lower clinical scores in MSC exosome-treated groups. All six studies (including ours) [23,24,25,26,27] showed reduced epidermal thickness following MSC exosome treatment. Different skin tissue cytokines were measured in the included studies. TNF-α and IL-17A were measured most often, with inconsistent results.

Moreover, three studies explored exosomes loaded with therapeutic cargos. Exosomes from mouse bone marrow-derived MSCs loaded with PD-L1 showed greater improvements in clinical severity, epidermal thickness, and cytokine suppression compared to unmodified exosomes [27]. hUCMSC exosomes loaded with ASO210 reduced skin thickness and cytokine levels, although the clinical scores were similar to those in the unmodified exosome group [25]. Exosomes loaded with the arginase-1 inhibitor nor-NOHA led to a thinner epidermis and more pronounced suppression of the nuclear factor-κB signaling pathway compared to unmodified exosomes [26]. No adverse events were reported in either the murine studies or the human trial.

#### 3.2.4. Meta-Analyses

The meta-analyses evaluated the effects of MSC exosomes on clinical severity scores, epidermal thickness, and cytokine levels in skin tissues, based on data from seven murine experiments (including the present study) [22,23,24,25,26,27]. In the study by Zhang et al. [22], two models, mild and moderate psoriasis, were used, but only data from the moderate model were included, as it was more consistent with the other studies.

**Figure 4:**
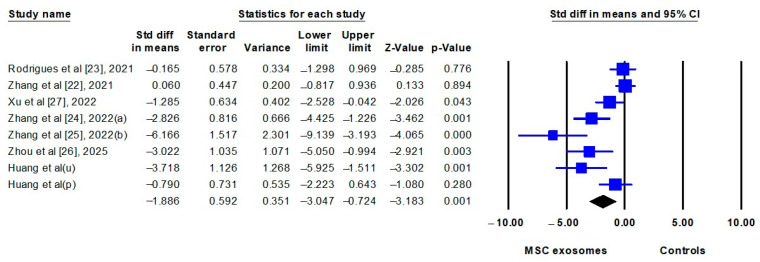
Forest plot comparing the clinical severity scores of imiquimod-induced psoriasis-like dermatitis in mice treated with mesenchymal stem cell exosomes (MSC exosomes) versus vehicle or no-treatment controls (controls). p, placenta; u, umbilical cord. The data of Huang et al. were from current study.

**Figure 5:**
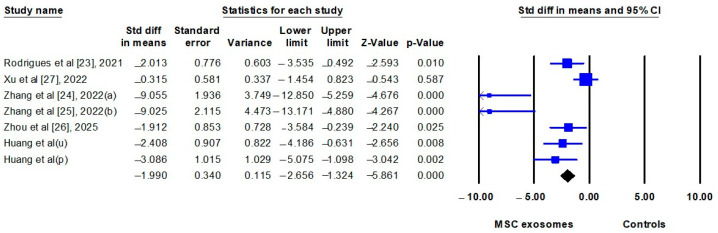
Forest plot comparing the epidermal thickness of imiquimod-induced psoriasis-like dermatitis in mice treated with mesenchymal stem cell exosomes (MSC exosomes) versus vehicle or no-treatment controls (controls). p; placenta; u, umbilical cord. The data of Huang et al. were from current study.

The results of the meta-analyses before and after incorporating the present study are summarized in Table 4. The pooled estimates were calculated by comparing IMQ-induced mice treated with MSC exosomes (exosome group) to IMQ-induced mice treated with vehicle controls or left untreated (control group). All seven studies (including the present study) [22,23,24,25,26,27] reported clinical severity scores, which were significantly lower in the exosome group (SMD: −1.886; 95% CI: −3.047 to −0.724) (Figure 4). Epidermal thickness was assessed in six studies (including the present study) [23,24,25,26,27] and was also significantly reduced in the exosome group (SMD: −3.258; 95% CI: −4.987 to −1.529) (Figure 5). Notably, one of the six studies provided data on skin thickness rather than epidermal thickness [25], but these data were pooled in this meta-analysis.

**Figure 6:**
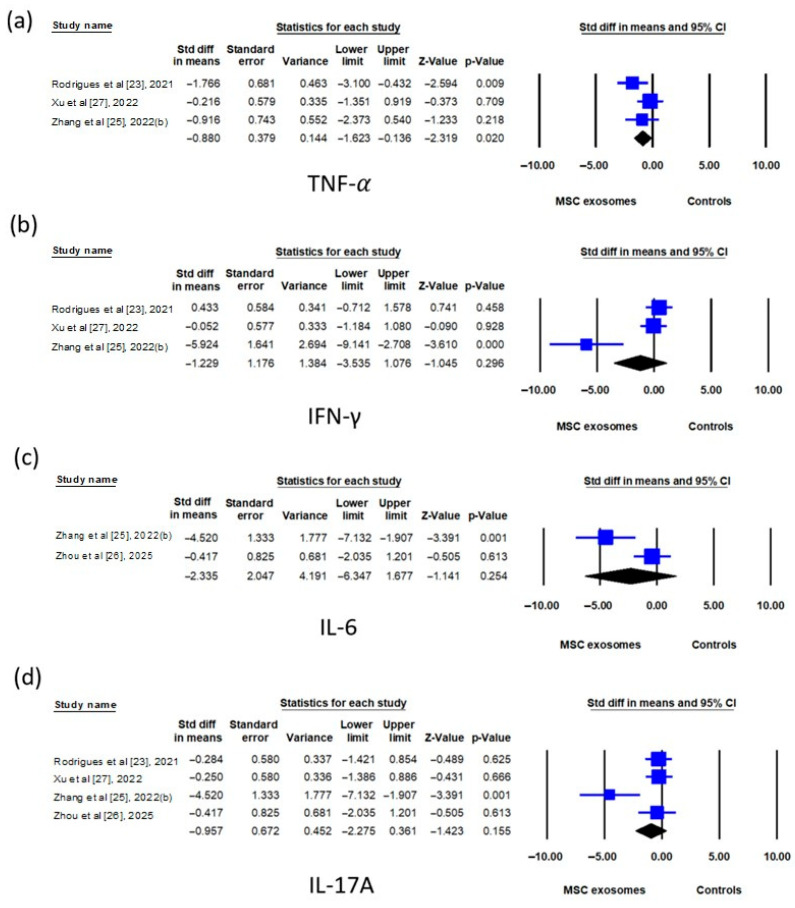
Forest plots comparing cytokine mRNA levels, namely, (**a**) TNF-α, (**b**) INF-γ, (**c**) IL-6, and (**d**) IL-17A, in the skin tissue of imiquimod-induced psoriasis-like dermatitis mice treated with mesenchymal stem cell exosomes (MSC exosomes) versus vehicle or no-treatment controls (controls). IFN-γ, interferon-γ; IL, interleukin; TNF-α, tumor necrosis factor-α.

**Figure 7:**
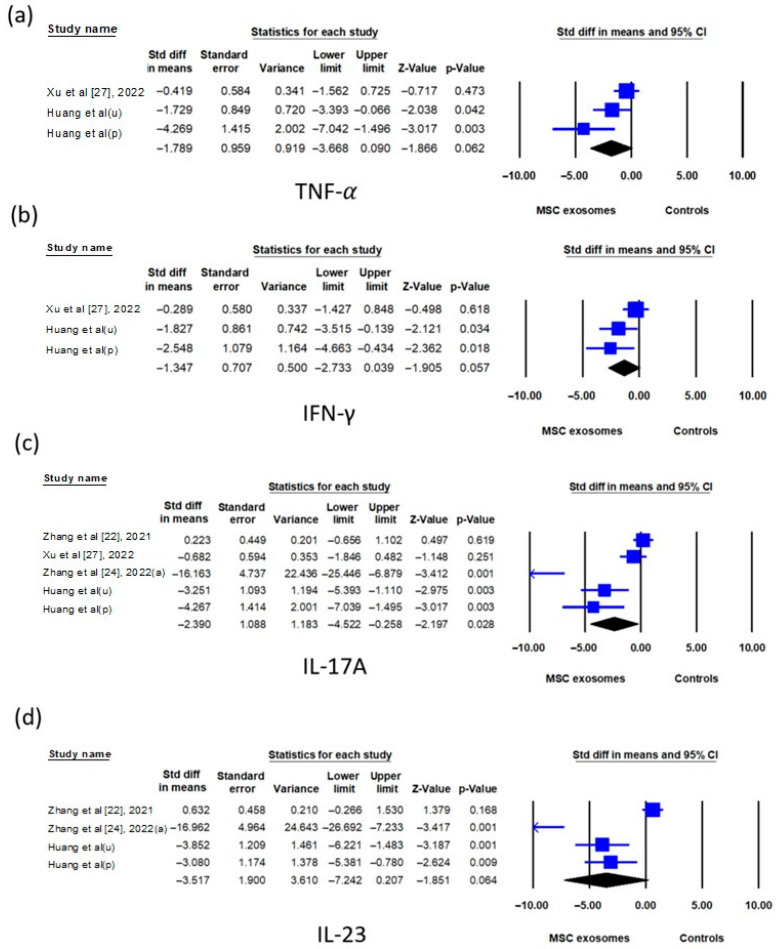
Forest plots comparing cytokine protein levels, namely, (**a**) TNF-α, (**b**) INF-γ, (**c**) IL-17A, and (**d**) IL-23, in the skin tissue of imiquimod-induced psoriasis-like dermatitis mice treated with mesenchymal stem cell exosomes (MSC exosomes) versus vehicle or no-treatment controls (controls). p, placenta; u, umbilical cord; IFN-γ, interferon-γ; IL, interleukin; TNF-α, tumor necrosis factor-α. The data of Huang et al. were from current study.

Regarding cytokine expression in skin tissues, the TNF-α mRNA level was significantly lower in the exosome group compared to the control group (SMD: −0.880; 95% CI: −1.623 to −0.136) (Figure 6a), whereas the TNF-α protein level showed only a trend toward reduction (SMD: −1.789; 95% CI: −3.668 to 0.090) (Figure 7a). No significant differences were found in the IFN-γ, IL-6, or IL-17A mRNA levels between the two groups (Figure 6b–d). However, the IL-17A protein level was significantly lower in the exosome group (SMD: −2.390; 95% CI: −4.522 to −0.258) (Figure 7c). The IFN-γ and IL-23 protein levels showed a trend toward reduction in the exosome group but did not reach statistical significance (Figure 7b or Figure 7d).

**Figure 8:**
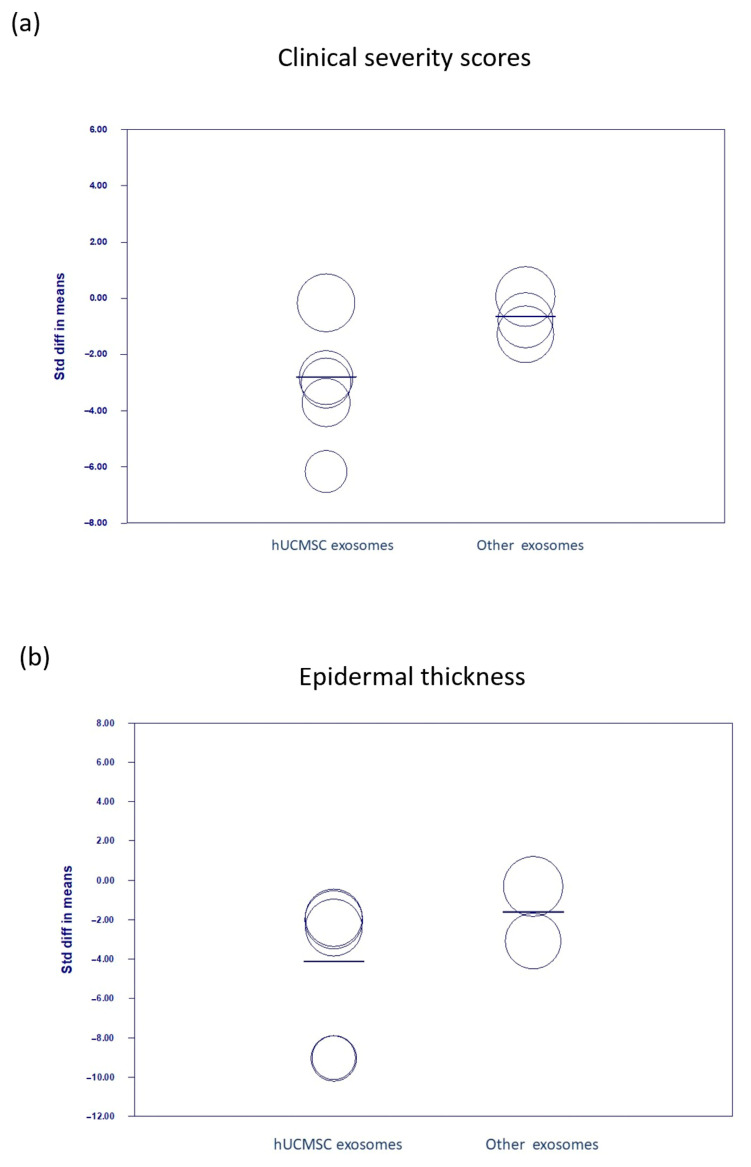
Scatter plot. The meta-regression compares the effectiveness of exosomes in improving (**a**) clinical severity scores (*p* = 0.030) and (**b**) epidermal thickness in studies with different MSC exosomes (*p* = 0.101). hUCMSC, human umbilical cord mesenchymal stem cell; std, standardized.

Most of the included studies used exosomes derived from hUCMSCs. A meta-regression analysis was conducted to compare the treatment effects of hUCMSC-derived exosomes versus those from other MSC sources. The scatter plot showed significant improvements in the clinical severity scores of the studies using hUCMSC exosomes (*p* = 0.030) (Figure 8a), whereas the improvements in epidermal thickness were comparable between the two groups (*p* = 0.101) (Figure 8b). Notably, publication bias was not assessed because of the limited number of included studies.

### 3.3. Comparing the Current Experimental Study with Meta-Analyses

Consistent with the meta-analysis results obtained before incorporating the current data, this experimental study demonstrated that MSC exosomes significantly improved clinical severity scores and epidermal thickness. After including the current study, these improvements became even more pronounced. This study determined cytokine protein levels in skin tissues. All cytokines showed a significant reduction in skin tissues in this study. After incorporating these data, both TNF-α and INF-γ were meta-analyzed, and IL-17A exhibited a significant improvement. The current study found that hUCMSCs and hPMSCs were similarly effective in mitigating psoriasis. However, with the addition of these data, there were sufficient studies to conduct a meta-regression, revealing that hUCMSCs outperformed other sources of MSC exosomes in enhancing clinical severity scores.

## 4. Discussion

Our experiments evaluated the effectiveness of MSC exosomes in treating psoriasis. The hPMSC and hUCMSC exosomes both showed better effectiveness in reducing epidermal thickness and skin tissue cytokines than the controls, and no significant difference was found between the two MSC exosomes. The results of the meta-analyses that included the current study indicated that MSC exosomes significantly improved both the clinical skin severity scores and epidermal thickness of psoriasis-affected skin. Additionally, the mRNA levels of TNF-α in skin tissue were reduced following treatment with MSC exosomes, although the protein levels remained unchanged. The protein levels of IL-17A were also reduced. Exosomes from hUCMSCs exhibited better efficacy in improving clinical severity scores than exosomes from other MSC sources.

Two studies [22,23] that applied topical MSC exosomes showed no significant difference in clinical severity scores in comparison to vehicle controls (gel and cream). Applying vehicles (gel and cream) alone may alleviate psoriatic symptoms. Further studies are needed to draw definitive conclusions. However, the epidermal thickness in the exosomes group was thinner than that of the vehicle control group. Based on the data from the in vitro studies, the possible mechanisms underlying the improvement in psoriasis caused by MSC exosomes may involve inhibiting T cell proliferation [23,25,27], inhibiting Th1 and Th17 differentiation [23,26], and inducing Treg [23,27] by influencing FOXP3 expression. MSC exosomes also suppressed DC maturation and activation [24,26] and inhibited the release of pro-inflammatory cytokines (TNF-α, IFN-γ, IL-1β) [20,23,26], chemokines (CCL20 and CXCL8) [23,24], and psoriasis-specific cytokines (IL-23) [24].

Other possible mechanisms were also unveiled in the murine studies. Zhang et al. demonstrated that topically applied exosomes derived from embryonic stem cells could effectively reduce the levels of IL-17A and the terminal complement activation complex C5b-9 in IMQ-induced mice models of mild psoriasis. The exosomes were found to inhibit complement activation within the stratum corneum, specifically by decreasing the formation of C5b-9 complexes mediated by CD59. This intervention alleviated the accumulation of neutrophils both within and beneath the stratum corneum, thereby reducing the release of IL-17A through neutrophil extracellular traps [22]. Another study demonstrated that subcutaneous injection of human umbilical cord MSC exosomes significantly decreased psoriasis-specific cytokines, such as IL-17A and IL-23, and inhibited the phosphorylation of STAT3 [24].

Another factor to consider is the complex composition of MSC exosomes; however, the precise components responsible for their biological functions are not yet fully understood. These exosomes contain a variety of elements derived from MSCs, such as proteins, lipids, DNA, mRNAs, and microRNAs (miRNAs). Among these, miRNAs have drawn significant interest due to their critical role in regulating immune responses [39]. Over 250 miRNAs have been identified as differentially expressed in the skin and blood of psoriasis patients [40]. Based on the data from our experiments, as well as from the meta-analyses results, exosomes from hUCMSCs showed better therapeutic potential in improving clinical severity than exosomes from other MSC sources. The diversity of miRNAs in different MSC exosomes may account for their varying efficacy in treating psoriasis. Despite considerable interest, the specific mechanisms of action and the principal therapeutic factors in MSC exosomes for psoriasis treatment remain largely unknown and require further investigation.

In addition to directly using MSC exosomes as a therapy for psoriasis, another approach involves encapsulating therapeutic drugs within exosomes to boost drug efficacy. One promising agent, ASO-210, has demonstrated effectiveness in correcting immune imbalances and the pathological microenvironment in psoriasis [41]. However, ASO-210 suffers from low stability and inefficient cell targeting [41]. By loading ASO-210 into exosomes, these issues are mitigated, and the drug properties are significantly enhanced, showcasing MSC exosomes as promising drug delivery tools [25]. Another noteworthy strategy uses exosomes derived from MSCs that are genetically modified via lentivirus-mediated gene transfection to overexpress PD-L1 [27]. These modified MSC exosomes have chemokine receptors that respond to chemokines in inflamed areas [42]. The increased vascular permeability of inflamed tissues allows fluids, macromolecules, and exosomes to easily reach and treat affected areas, leaving the bloodstream through small vessels [43]. In vivo data revealed that PD-L1-overexpressing MSC exosomes could recognize various activated immune cells, including T cells, macrophages, and DCs, exhibiting high PD-1 expression, facilitating PD-1 and PD-L1 interactions in inflammation [27]. Additionally, a study reported the loading of an Arg1 inhibitor, nor-NOHA, into MSC exosomes. This combination profoundly suppressed the NF-κB signaling pathway by targeting the Arg1/polyamine-mediated DCs/Th17 axis. The approach scavenged self-antigens, resulting in superior alleviation of skin lesions and modulation of both local and systemic metabolic and immunological imbalances, surpassing the effects achieved by unmodified MSC exosomes [26].

This study has several limitations. First, we were only able to include one human trial, which lacked a control group, so further research is needed to evaluate the efficacy and safety of MSC exosomes. Despite this, several clinical trials have already examined the safety of intravenous MSC exosomes in treating conditions like respiratory distress syndrome and complex regional pain syndrome, with no reported adverse events or safety issues [44,45]. Second, our meta-analysis included seven murine experiments; thus, the limited number of studies and their relatively small sample sizes must be considered. Third, the studies we included varied in terms of MSC exosome types, concentrations, and treatment protocols, which may explain the high heterogeneity observed. Lastly, we only included individual data from our current study, so we cannot account for other potential confounding factors that could have influenced the outcomes. However, the murine studies were generally well-controlled.

In summary, MSC-derived exosomes effectively improve the clinical manifestations of psoriasis by modulating inflammatory pathways and exhibit a favorable safety profile. Among various sources, hUCMSC exosomes were the most extensively studied. However, direct comparative studies are required to validate the superior efficacy of hUCMSC exosomes compared with exosomes from other MSC origins. MSC exosomes also offer significant potential as nanocarriers for delivering therapeutic agents in psoriasis treatment. However, the precise molecular mechanisms and bioactive components of MSC exosomes from different sources remain to be fully elucidated. Future studies should focus on establishing standardized protocols for exosome isolation and characterization, as well as developing advanced engineering strategies to enhance their therapeutic specificity and efficacy. To validate their clinical utility, larger and well-designed randomized controlled trials in human populations are urgently needed.

## Figures and Tables

**Table 4:** The results of meta-analyses before and after incorporating the current study.

Outcomes	Study Included	Effect Size	Effect Estimate (95% CI)	*p*-Value	*I* ^2^
**Before incorporation**					
Clinical severity scores	6	SMD	−1.852 (−3.240 to −0.464)	0.009	82.3%
Epidermal thickness	5	SMD	−3.740 (−6.232 to −1.249)	0.003	84.6%
Cytokines in skin tissues					
mRNA level					
TNF-α	3	SMD	−0.880 (−1.623 to −0.136)	0.020	33.6%
IFN-γ	3	SMD	−1.229 (−3.535 to 1.076)	0.296	85.1%
IL-6	2	SMD	−2.335 (−6.347 to 1.677)	0.064	85.4%
IL-17A	4	SMD	−0.957 (−2.275 to 0.361)	0.155	67.9%
Protein level					
IL-17A	3	SMD	−1.175 (−3.593 to 1.244)	0.341	84.5%
IL-23	2	SMD	−7.471 (−24.659 to 9.718)	0.394	92.0%
**After incorporation**					
Clinical severity scores	7	SMD	−1.886 (−3.047 to −0.724)	<0.001	79.5%
Epidermal thickness	6	SMD	−3.258 (−4.987 to −1.529)	<0.001	82.4%
Cytokines in skin tissues					
mRNA level					
TNF-α	3	SMD	−0.880 (−1.623 to −0.136)	0.020	33.6%
IFN-γ	3	SMD	−1.229 (−3.535 to 1.076)	0.296	85.1%
IL-6	2	SMD	−2.335 (−6.347 to 1.677)	0.064	85.4%
IL-17A	4	SMD	−0.957 (−2.275 to 0.361)	0.155	67.9%
Protein level					
TNF-α	2	SMD	−1.789 (−3.668 to 0.090)	0.062	71.0%
IFN-γ	2	SMD	−1.347 (−2.733 to 0.039)	0.057	55.2%
IL-17A	4	SMD	−2.390 (−4.522 to −0.258)	0.028	85.0%
IL-23	3	SMD	−3.517 (−7.242 to 0.207)	0.064	89.9%

CI, confidence interval; IFN-γ, interferon-γ; IL, interleukin; SMD, standardized mean difference; TNF-α, tumor necrosis factor-α.

## Data Availability

Our manuscript includes data as electronic Supplementary Materials. Other data underlying this article will be shared upon reasonable request to the corresponding author.

## Supplementary Materials

The following supporting information can be downloaded at https://www.mdpi.com/article/10.3390/biomedicines13092093/s1, Table S1: The search strings in PubMed; Table S2: Extracted data of clinical severity scores and epidermis thickness; Table S3: Extracted data of mRNA and protein cytokine levels in skin tissues; Table S4: Risk of bias using the Systematic Review Centre for Laboratory Animal Experimentation (SYRCLE) tool for murine randomized controlled experiments; Table S5: Risk of bias using the Toxicological Data Reliability Assessment Tool (ToxRTool) for in vitro model studies. Figure S1: The change in concentration of exosomes stored at different temperatures over time. Figure S2: Representative results of immunoblotting showing exosomes derived from hPMSCs and hUCMSCs.

## Abbreviations

The following abbreviations are used in this manuscript:

ASO210	antisense oligonucleotides targeting miR-210
CI	confidence interval
DC	dendritic cell
ELISA	enzyme-linked immunosorbent assay
EVs	extracellular vesicles
hPMSC	human placenta-derived MSC
hUCMSC	human umbilical cord-derived MSC
IL	interleukin
IMQ	imiquimod
INF-γ	interferon-γ
miRNAs	microRNAs
MSC	mesenchymal stem cell
NTA	nanoparticle tracking analysis
nor-NOHA	arginase-1 inhibitor
PASI	psoriasis area and severity index
PD-L1	programmed cell death-ligand 1
PBMCs	peripheral blood mononuclear cells
sEVs	small extracellular vesicles
SMD	standardized mean difference
SYRCLE	Systematic Review Centre for Laboratory Animal Experimentation
TEM	transmission electron microscopy
TNF-α	tissue necrosis factor-α
ToxRTool	Toxicological Data Reliability Assessment Tool
Treg	regulatory T cell
