# Supplementary material for: Effectiveness of Exosomes from Different Mesenchymal Stem Cells in the Treatment of Psoriasis: A Murine Study and Meta-Analysis of Experimental Studies
Source: Biomedicines. 2025 Aug 28;13(9):2093. doi: 10.3390/biomedicines13092093 (PMC12467663; doi:10.3390/biomedicines13092093)

SUPPLEMENTERY INFORMATIONS

Table S1. The search strings in PubMed

Search number	Query	Search Details	Results
5	(#1) AND (#4)	("psoriasis"[MeSH Terms] OR "psoriasis"[All Fields] OR "psoriases"[All Fields] OR "psoriasi"[All Fields]) AND ("exosomal"[All Fields] OR "exosomes"[MeSH Terms] OR "exosomes"[All Fields] OR "exosome"[All Fields] OR "exosomic"[All Fields] OR ("extracellular vesicles"[MeSH Terms] OR ("extracellular"[All Fields] AND "vesicles"[All Fields]) OR "extracellular vesicles"[All Fields] OR ("extracellular"[All Fields] AND "vesicle"[All Fields]) OR "extracellular vesicle"[All Fields]))	122
4	(#2) OR (#3)	"exosomal"[All Fields] OR "exosomes"[MeSH Terms] OR "exosomes"[All Fields] OR "exosome"[All Fields] OR "exosomic"[All Fields] OR ("extracellular vesicles"[MeSH Terms] OR ("extracellular"[All Fields] AND "vesicles"[All Fields]) OR "extracellular vesicles"[All Fields] OR ("extracellular"[All Fields] AND "vesicle"[All Fields]) OR "extracellular vesicle"[All Fields])	64,578
3	extracellular vesicles	"extracellular vesicles"[MeSH Terms] OR ("extracellular"[All Fields] AND "vesicles"[All Fields]) OR "extracellular vesicles"[All Fields] OR ("extracellular"[All Fields] AND "vesicle"[All Fields]) OR "extracellular vesicle"[All Fields]	51,730
2	exosomes	"exosomal"[All Fields] OR "exosomes"[MeSH Terms] OR "exosomes"[All Fields] OR "exosome"[All Fields] OR "exosomic"[All Fields]	36,812
1	psoriasis	"psoriasis"[MeSH Terms] OR "psoriasis"[All Fields] OR "psoriases"[All Fields] OR "psoriasis"[All Fields]	69,890

Table S2. Extracted data of clinical severity scores and epidermis thickness

Study	Group	Sample size	Clinical score (PASI)	Epidermal thickness (μm)
Rodrigues et al[23], 2021	IMQ+vehicle	6	2.83±0.936[a]	66.129±14.516
	IMQ+UCB-MNC-sEVs	6	2.702±0.574[a]	43.871±5.806
Zhang et al[22], 2021	IMQ+vehicle	10	8.639±1.485	NA
	IMQ+MSC-Exo	10	8.721±1.258	NA
Xu et al[27], 2022	IMQ	6	5.352±1.173[b]	23.579±13.923
	IMQ+MSC-sEVs	6	3.972±0.965[b]	20±7.993
Zhang et al[24], 2022(a)	IMQ+PBS	6	7.508±1.261	75±5
	IMQ+MSC-Exo	6	4.154±1.108	23.333±6.334
Zhang et al[25], 2022(b)	IMQ+PBS	5	8.794±1.313	2.103±0.147[c]
	IMQ+ MSC-IFNγ-sEVs	5	2.534±0.581	1.11±0.051[c]
Zhou et al[26], 2025	IMQ+PBS	4	7.875±1.063	146.588±28
	IMQ+MSC-EVs	4	4±1.469	87.294±33.765
Huang et al	IMQ+vehicle	6	4.50±0.5	46.021±6.717
	IMQ+MSC-Exo (P)	6	3.833±0.687	35.628±2.835
	IMQ+MSC-Exo (U)	6	3.167±0.373	31.423±4.661

Exo, exosome; IMQ, imiquimod; IFNγ-sEVs, IFNγ stimulated sEVs; IL, interleukin; MNC, mononuclear cell; MSC, mesenchymal stem cell; PASI, psoriasis area and severity index; PBS, phosphate buffered saline; sEV, small extracellular vesicles; UVB, umbilical cord blood

All data are presented as mean ± standard deviation

[a]clinical score is presented as PASI (ranging from 0 to 12) but the score in this study ranges from 0 to 10

[b]the standard deviation is calculated from the standard error

[c]skin thickness (mm)

Table S3. Extracted data of mRNA and protein cytokine levels of skin tissues

Study	Group	Sample size	mRNA (fold change)				Protein			
			TNF-α	IFN-γ	IL-6	IL-17	TNF-α	IFN-γ	IL-17	IL-23
Rodrigues et al[23], 2021	IMQ+vehicle	6	1.837± 0.612	1.656± 1.669	NA	1.231± 0.538	NA	NA	NA	NA
	IMQ+UCB-MNC-sEVs	6	0.918± 0.409	2.212± 0.715	NA	1.051± 0.718	NA	NA	NA	NA
Zhang et al[22], 2021	IMQ+vehicle	10	NA	NA	NA	NA	NA	NA	100± 43.111[c]	100± 20.889[c]
	IMQ+MSC-Exo	10	NA	NA	NA	NA	NA	NA	110.222± 48.445[c]	114.222± 24[c]
Xu et al[27], 2022	IMQ	6	134.848± 76.703[a]	150± 83.109[a]	523.077± 370.564[a]	203.774± 150.205[a]	118.511± 62.54[d]	75.484± 31.079[d]	222.078± 82.709[d]	NA
	IMQ+MSC-sEVs	6	119.697± 63.094[a]	145.833± 77.277[a]	487.179± 471.054[a]	175.472± 55.461[a]	98.085± 29.186[d]	65.376± 38.455[d]	166.234± 81.12[d]	NA
Zhang et al[24], 2022(a)	IMQ+PBS	6	NA	NA	NA	NA	NA	NA	0.937± 0.036[e]	1.005± 0.032[e]
	IMQ+MSC-Exo	6	NA	NA	NA	NA	NA	NA	0.477± 0.018[e]	0.495± 0.028[e]
Zhang et al[25], 2022(b)	IMQ+PBS	6	1±0.425	0.941± 0.134	1.061± 0.112	0.946± 0.092	NA	NA	NA	NA
	IMQ+ MSC-IFNγ-sEVs	6	0.712± 0.13	0.274± 0.086	0.388± 0.023	0.495± 0.107	NA	NA	NA	NA
Zhou et al[26], 2025	IMQ+PBS	4	NA	NA	NA	1.364± 0.901[b]	NA	NA	NA	NA
	IMQ+MSC-EVs	4	NA	NA	NA	1.049± 0.574[b]	NA	NA	NA	NA

Huang et al	IMQ+vehicle	5	NA	NA	NA	NA	80.926± 8.738[f]	79.044± 2.811[f]	9.979± 0.971[f]	53.600± 1.982[f]
	IMQ+MSC-Exo(P)	5	NA	NA	NA	NA	62.942± 3.671[f]	68.704± 5.207[f]	7.215± 0.614[f]	34.875± 7.488[f]
	IMQ+MSC-Exo(U)	5	NA	NA	NA	NA	60.694± 4.97[f]	70.734± 4.36[f]	7.369± 0.614[f]	34.002± 5.751[f]

Exo, exosome; IMQ, imiquimod; IFN, interferon; IFNγ-sEVs, INFγ stimulated sEVs; MNC, mononuclear cell; MSC, mesenchymal stem cell; NA, not available; P, placenta; PBS, phosphate buffered saline; sEV, small extracellular vesicles; TNF, tumor necrosis factor; U, umbilical cord; UVB, umbilical cord blood

All data are presented as mean ± standard deviation; the standard deviation is calculated from the standard error in the study of Xu et al.

a results from qPCR, relative to β-actin

b results from qPCR, relative to GAPDH

c results from ELISA, relative % to vehicle

d pg/100mg protein

e results from Western blot, relative to GAPDH

f pg/ml

Table S4. Risk of bias using the Systematic Review Centre for Laboratory Animal Experimentation (SYRCLE) tool for murine randomized controlled experiments.

Study	Random sequence generation	Baseline characteristics	Allocation concealment	Random animal housing	Blinding of participants and personnel	Random selection for outcome assessment	Blinding of outcome assessment	Incomplete outcome data	Selective outcome reporting	Other bias
Rodrigues et al[23], 2021	UC	L	UC	UC	UC	UC	UC	L	L	L
Zhang et al[22], 2021	UC	L	UC	UC	UC	UC	UC	L	L	L
Xu et al[27], 2022	UC	L	UC	UC	UC	UC	UC	L	L	L
Zhang et al[24], 2022(a)	UC	L	UC	UC	UC	UC	UC	L	L	L
Zhang et al[25], 2022(b)	UC	L	UC	UC	UC	UC	UC	L	L	L
Zhou et al[26], 2025	UC	L	UC	UC	UC	UC	UC	L	L	L
Huang et al	L	L	L	L	H	L	L	L	L	L

H, high risk of bias; L, low risk of bias; UC, unclear risk of bias

Table S5. Risk of bias using the Toxicological data Reliability Assessment Tool (ToxRTool) for in vitro model studies.

Study	Group I: Test substance identification	Group II: Test system characterization	Group III: Study design description	Group IV: Study results documentation	Group V: Plausibility of study design and data	Total	Initial category[a] (based on combined score)	Revised category (after checking red criteria)
Rodrigues et al[23], 2021	4	3	6	3	2	18	1	1
Xu et al[27], 2022	4	3	5	3	2	17	1	3[b]
Zhang et al[24], 2022(a)	4	3	6	3	2	18	1	1
Zhang et al[25], 2022(b)	4	3	5	3	2	17	1	3[b]
Kim et al[20], 2023	4	3	6	3	2	18	1	1
Abed et al[21], 2024	3	3	5	3	1	15	1	3[c]
Zhou et al[26], 2025	4	3	6	3	2	18	1	1

a Category 1: reliable without restrictions; Category 2: reliable with restrictions; Category 3: not reliable

b The concentration of exosomes is unclear

c The source of MSC exosomes is unclear

Figure S1. The change in concentration of exosomes stored in different temperatures over time. X-axis: days; Y-axis: particles/mL

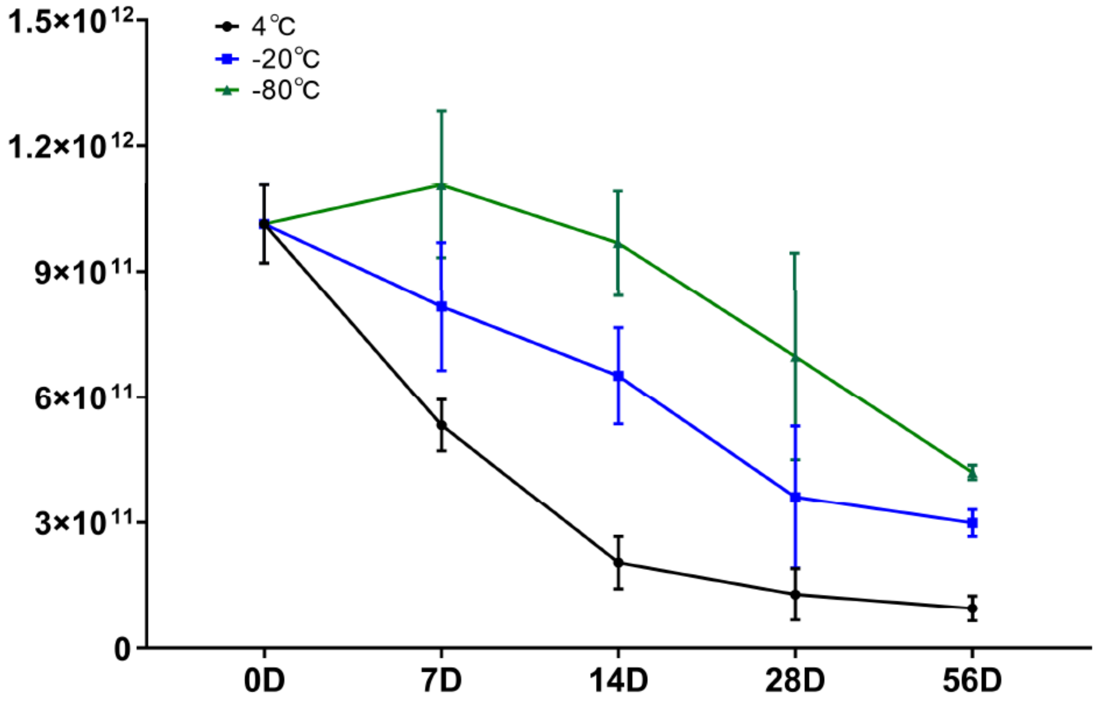

Figure S2. Representative results of immunoblotting showing exosomes derived from hPMSCs and hUCMSCs. The red boxes indicate the full-length blots of each protein.

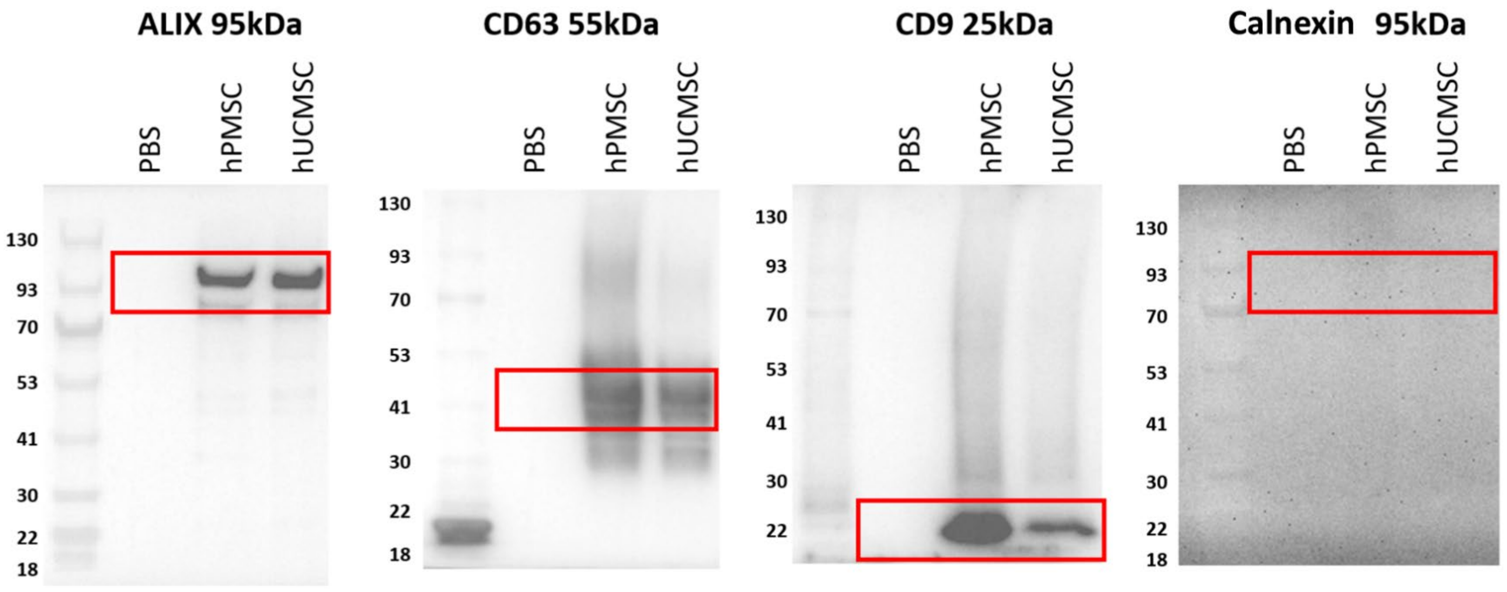

Supplement: Supplementary file 1 [file biomedicines-13-02093-s001.zip › biomedicines-3817370-supplementary.pdf]
